# Fatigue Models for Airfield Concrete Pavement: Literature Review and Discussion

**DOI:** 10.3390/ma14216579

**Published:** 2021-11-02

**Authors:** Jie Yuan, Wenhao Li, Yuening Li, Lukuan Ma, Jiake Zhang

**Affiliations:** 1 Key Laboratory of Road and Traffic Engineering of the Ministry of Education, Tongji University, Shanghai 201804, China; yuanjie@tongji.edu.cn (J.Y.); li_wenhao@tongji.edu.cn (W.L.); 2 Key Laboratory of Infrastructure Durability and Operation Safety in Airfield of CAAC, Tongji University, Shanghai 201804, China; 3 School of Civil Engineering, University of Sydney, Sydney, NSW 2006, Australia; winnieli321@163.com

**Keywords:** airfield, concrete pavement, fatigue model, slab thickness, improvement method

## Abstract

The fatigue model plays an important role in the mechanistic–empirical design procedure of airfield pavement. As for cement concrete pavement, the fatigue model represents the relationship between the stress and the number of load repetitions. To further understand the fatigue model, a literature review was performed in this paper along with the discussion. In this paper, the developed fatigue models available now were classified as the full-scale testing-based fatigue model and the concrete beam testing-based fatigue model, according to the data source. Then, the regression analysis process and stress calculation method of each fatigue model were summarized. Besides, the fatigue model proposed by the Federal Aviation Administration (FAA) was compared with the fatigue model of the Civil Aviation Administration of China (CAAC). The design thicknesses using the two models were obtained based on the finite element analysis. The results show that the designed slab using the fatigue model of FAA is thicker than that of CAAC, meaning that the fatigue model of FAA is comparatively conservative. Moreover, it can be concluded that the differences in the slab thickness become more significant with the increase in the wheel load and the foundation strength. Finally, the recommendation was proposed to refine the fatigue model in the future study from three aspects: data source, stress calculation method, and regression analysis process.

## 1. Introduction

Cement concrete pavement is a common structural type in the airfield. Under mechanical loading, the concrete slab often experiences structural damage while the stress is far below the ultimate strength of the concrete slab. This kind of damage is the fatigue cracking caused by repeated loading. The development of fatigue cracking deteriorates the pavement performance, which has a detrimental influence on the service life of the pavement structure. Therefore, it is necessary to ensure that the pavement structure has sufficient thickness to resist fatigue cracking. In the mechanistic–empirical design procedure of airfield pavement, it is essential to calculate the ultimate load repetitions to the fatigue failure of concrete slabs. The basis of the calculation is the fatigue model that describes the relationship between the stress of the concrete slab induced by load and the number of load repetitions [1,2,3,4]. In practice, the fatigue model for airfield pavements plays an important role, not only in the design of pavement structure, but also in the evaluation of the remaining service life for in-service pavements [5]. 

The fatigue mechanism of concrete pavement is complicated, because the pavement performances are affected by the pavement structure, the surrounding environment, the wheel loading, etc. [6,7]. The fatigue mechanism reflected by the mechanistic model of the concrete structure is different from that of the pavement structure in service. Thus, the fatigue models for airfield pavements are commonly developed based on the regression analysis of the fatigue test data. Accordingly, the fatigue models are generally divided into two types: one is proposed based on the on-site full-scale test data, and the other is developed based on the laboratory concrete beam test data. Due to the difference in test data and regression analysis processes, the parameters of the fatigue models are usually different.

As for airfield concrete pavement, the fatigue model is associated with the stress calculation theory and critical stress location [8]. In the design of airfield concrete pavement, it is common that the designed thicknesses are obviously different due to different fatigue models. It is difficult for the designers to make a trade-off between the reasonable structural design and the economy [9]. Therefore, the development process and critical mechanism of the fatigue model for airfield concrete pavements are reviewed in this paper. Subsequently, the thickness differences based on typical fatigue models in the current airfield concrete pavement design method are analyzed. Moreover, this paper proposes how to improve the fatigue model of airfield concrete pavements in the future. 

## 2. Literature Review of Fatigue Models of Airfield Concrete Pavements

### 2.1. Full-Scale Testing-Based Fatigue Models

**Figure 1:**
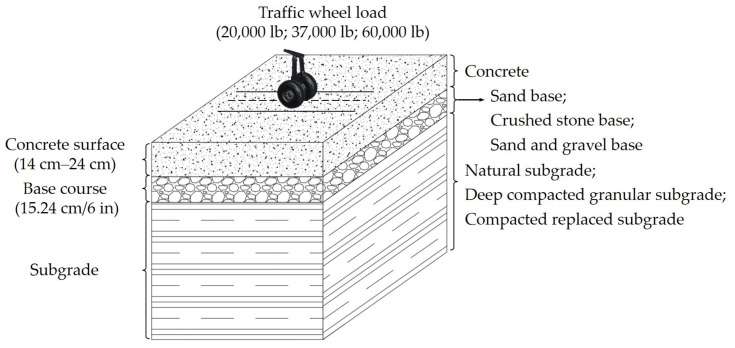
The structure and loading of the full-scale test at Lockbourne.

The early fatigue model was proposed by the USA Army Corps of Engineers (COE). In the 1940s, COE proposed the fatigue model for airfield concrete pavement based on the full-scale test data at Lockbourne and the Westergaard edge stress theory [10,11]. The pavement structure information and traffic loading for Lockbourne No.1 test sections are shown in Figure 1 [12]. COE assumed that the concrete slab can withstand 5000 coverages to satisfy the service life [13]. The fatigue failure of concrete slab was defined as 50 percent of the slabs cracking. The fatigue model is shown as follows [14]:

(1) $$DF=\frac{MR}{0.75\sigma_{e}}=1.3$$

where:

DF is the design factor;
MR is the concrete modulus of rupture, which is equal to the flexural strength of concrete;
$\sigma_{e}$ is the edge stress calculated by the Westergaard stress theory;
0.75 is a stress reduction coefficient.

In the fatigue model of COE, the design factor (DF) was defined as the ratio of the concrete modulus of rupture to the stress at the edge of the slab. The DF of 1.3 was determined to consider factors affecting fatigue failure, such as the effect of loading, thermal curling, and the support of the base layer and the foundation [15]. The service life of the concrete slab can be obtained when the design factor is greater than 1.3.

The fatigue model of COE indicated that the calculated stress at the bottom of the concrete slab was the maximum stress multiplied by 0.75, which considers the effect of the joint load transfer. The effect of joint load transfer on stress reduction proposed by COE had been adopted by the subsequent researches. Although the formula and parameters of the fatigue model proposed by COE provided guidance for the follow-up research, it was oversimplified for the complex pavement condition.

Then, COE made efforts to improve the initial fatigue model. The pavement thickness designed for 5000 coverages was regarded as the criterion. The segmented fatigue models for the pavement structure with different service life were proposed, as shown in Equations (2) and (3) [16]:

(2) RH=1+0.07058×(logC−3.69897) for C < 5000

(3) RH=1+0.15603×(logC−3.69897) for C > 5000

where:

RH is the relative thickness factor;
C is the coverage to failure.

Compared with the initial fatigue model, this fatigue model established the relationship between the coverage and the relative design thickness of the concrete slab. The slope of the fatigue curve of the pavement structure with more than 5000 coverages had been adjusted, instead of simplifying the influence of various factors to 1.3 [17,18]. The adjusted fatigue model had a broader scope of application and fitted better with the real pavement structure.

In 1979, COE systematically reanalyzed the full-scale test data from 1943 to 1973, adopting the layered elastic analysis approach used in pavements. The modified fatigue model is shown as follows [6,19]:

(4) $$DF_{LEA}=\frac{MR}{\sigma_{LEA}}=0.58901+0.35486\times \log_{10}C$$

where:

$DF_{LEA}$ is the design factor based on layered elastic analysis;
MR is the modulus of rupture;
$\sigma_{LEA}$ is the maximum principal tensile stress at the bottom of the concrete slab based on layered elastic analysis;
C is the coverage to failure.

In Equation (4), the empirical relationship between the critical stress at the bottom of the concrete slab and the number of the coverages to failure was developed for the first time. The results showed that the design factor is linear with the logarithmic value of coverage. With the increase in the service life of the airport pavement, the critical coverages to fatigue increased. When the number of coverages to fatigue failure was increased, the design factor calculated by the fatigue model was also increased. Then, a thicker slab is needed to satisfy the design requirements.

**Figure 2:**
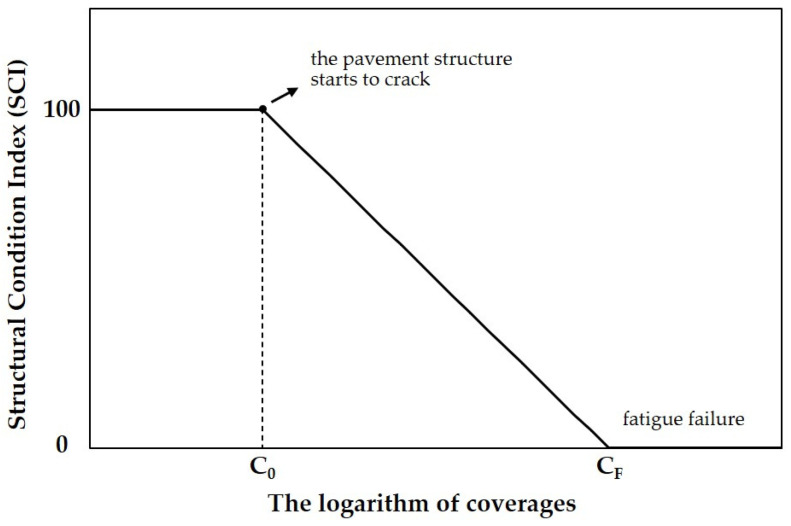
The relationship between SCI and the logarithm of coverages.

The subsequent fatigue models were mainly obtained by improving the fatigue model proposed by COE in 1979. In 1988, Rollings proposed the Structural Condition Index (SCI) to characterize the damage of pavement structure. Compared to the Pavement Condition Index (PCI), SCI was deducted by structural distresses induced by load, and the distresses resulted from the non-load case were ignored. It was found that the SCI deteriorates as a linear function of the logarithm of coverages, as shown in Figure 2. The modified fatigue model based on the definition of SCI is shown as follows [20]:

(5) $$SCI=\frac{DF-0.2967-\left(0.3881+0.000039\times SCI\right)\times \log_{10}C}{0.002269}$$

where:

SCI is the structural condition index.

Using Equation (5), the fatigue model based on the full-scale test data of COE from 1943 to 1973 was developed by the regression analysis of SCI, design factors, and coverages. When the value of SCI was different, the design factor and coverage had a different regression relationship. In fact, the SCI of 80 was equivalent to the fatigue failure of pavement structure assumed by COE. The form of the fatigue model with 80 of SCI was the same as that proposed by COE in 1979, which is shown as follows:

(6) $$DF=0.4782+0.3912\times \log_{10}(C_{80})$$

where:

DF is the design factor (MR/$\sigma_{LEA}$);
$C_{80}$ is the coverage to an SCI of 80.

Furthermore, Darter recalculated the stresses in the full-scale test data of COE from 1943 to 1979 by the H-51 computer program instead of the Westergaard edge stress theory. The H-51 program can calculate the stresses quickly by implementing the Pickett and Ray influence charts [21,22]. The exponential fatigue model was proposed by Darter, as shown in Equation (7) [23]. The fatigue model of Darter was limited by the assumption of the infinite slab in the H-51 computer program. It was also a lack of the consideration of the temperature curling influence.

(7) $$\log_{10}N=2.13{\left(\frac{MR}{\sigma_{e}}\right)}^{1.2}$$

where:

N is the number of coverages for 50 percent of the slabs cracking;
$\sigma_{e}$ is the critical edge stress calculated by H-51.

Another important fatigue model was developed by Foxworthy according to the full-scale test data conducted by COE, as expressed in Equation (8). To obtain the stresses closer to the real pavement condition, Foxworthy reanalyzed the COE test data by the ILLI-SLAB finite element program [24]. Compared with other fatigue models, the fatigue model proposed by Foxworthy was more conservative, especially in a high level of coverages. Although Foxworthy conducted a comprehensive analysis of the slab stresses to develop the pavement evaluation method, the influence of temperature curling on the slab stresses was also not considered.

(8) $$\log_{10}N=1.323\left(\frac{MR}{\sigma }\right)+0.588$$

where:

N is the number of coverages for 50 percent of the slabs cracking;
σ is the critical edge stress calculated by ILLI-SLAB.

For all the improved fatigue models, the stress calculation method varied from the Westergaard edge stress theory method to computer calculation procedures, while the essence of the fatigue model remained almost the same. However, the fatigue models were affected by the limitations of the full-scale test conducted by COE, such as the location of the test site and the loading conditions. Moreover, the effects of the temperature and environmental factors, which contributed to the fatigue characteristics of the real pavement structure, were ignored. The limitations above resulted in the difference between the theoretical calculation and the real structure. Considering the limitation, in 1992, Thompson and Barenberg proposed the NCHRP 1-26 fatigue model by recalculating the stresses in the full-scale test data of COE and the road test data of AASHO [25,26,27]. The NCHRP 1-26 fatigue model is shown as follows:

(9) $$\log_{10}N=-1.7136\left(\frac{\sigma }{MR}\right)+4.284\;\;\;\;\;\;\;\;\;\mathrm{for}\;\frac{\sigma }{MR}>1.25$$

(10) $$\log_{10}N=2.8127{\left(\frac{\sigma }{MR}\right)}^{-1.2214}\;\mathrm{for}\;\frac{\sigma }{MR}<1.25$$

where:

N is the number of coverages for 50 percent of the slabs cracking;
σ is the critical edge stress calculated by ILLI-SLAB.

The fatigue model comprehensively considered the effects of aircraft landing gear loading and wheel loading on the fatigue deterioration of concrete slabs. It also considered the influence of thermal curling on the slab stresses in the process of developing the fatigue model for the first time. Therefore, it was widely used in the airfield pavement design and highway pavement design due to the comprehensive analysis of the influencing factors of the fatigue characteristics of concrete slabs.

In recent years, the Federal Aviation Administration (FAA) has improved the fatigue model based on the research of COE. In the Federal Aviation Administration Rigid and Flexible Iterative Elastic Layer Design (FAARFIELD) released by FAA, the fatigue model is as follows [28]:

(11) $$DF=\frac{R}{0.75\sigma_{e}}=\left[\frac{F_{S}^{\prime }bd}{\left(1-\alpha \right)\left(b-d\right)+F_{S}^{\prime }b}\right]\times \log_{10}C+\left[\frac{\left(1-\alpha \right)\left(ad-bc\right)+F_{S}^{\prime }bc}{\left(1-\alpha \right)\left(b-d\right)+F_{S}^{\prime }b}\right]$$

(12) $$\alpha =\frac{SCI}{100}$$

where:

DF is the design factor;
$\sigma_{e}$ is the critical edge stress calculated by the FAARFIELD procedure;
R is the concrete flexural strength;
$F_{S}^{\prime }$ is the stabilization factor;
a, b, c, d are the regression coefficients;
a is 0.5878, b is 0.2523, c is 0.7409, d is 0.2465.

**Figure 3:**
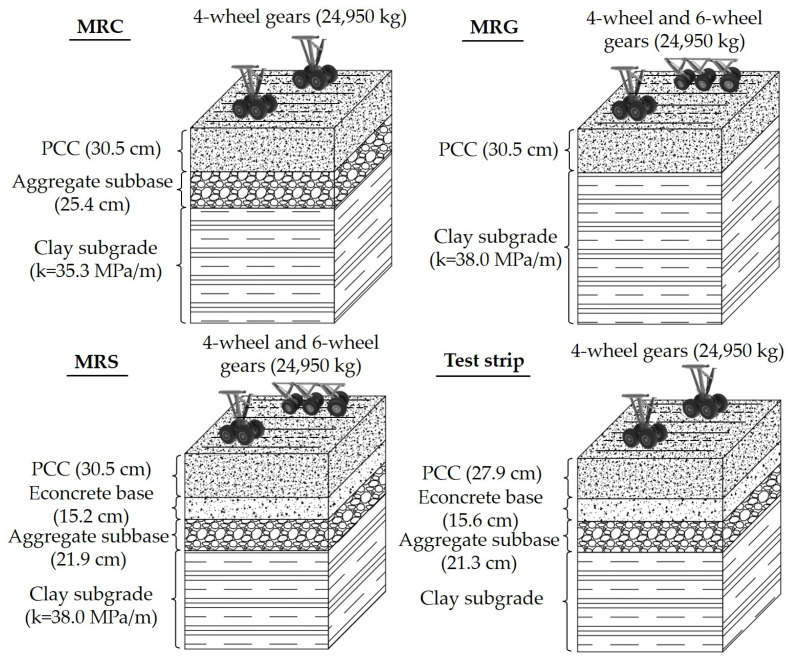
The pavement structure and loading method for the NAPTF CC2 test items.

FAA used the three-dimensional finite element software to recalculate the stresses in the historical full-scale test data conducted by COE [29,30]. The concept of SCI was adopted based on the study of Rollings. In addition, the new data points were supplemented from the National Airport Pavement Test Facility (NAPTF) Construction Cycle 2 (CC2) test. The CC2 test items, including MRC, MRG, MRS, were designed with different pavement structures and loading methods [31]. The information on the pavement structure and loading method for the test items as well as the test strip is shown in Figure 3 [31,32,33]. 

The test data from the test items and the test strip were supplemented to the historical full-scale database conducted by COE. The dataset for regression analysis in the development of this fatigue model consisted of the 30 data points derived from the historical full-scale test and 7 data points from NAPTF. The details of the data sources are shown in Table 1. Based on the research of Rollings [20], the relationship of SCI, DF, and coverages was reestablished, with consideration of the support of the base layer and foundation. Since the stresses calculated by the three-dimensional finite element software were closer to the real pavement structure condition, the fatigue model of FAA more accurately represented the fatigue characteristics.

### 2.2. Concrete Beam Testing-Based Fatigue Model

**Figure 4:**
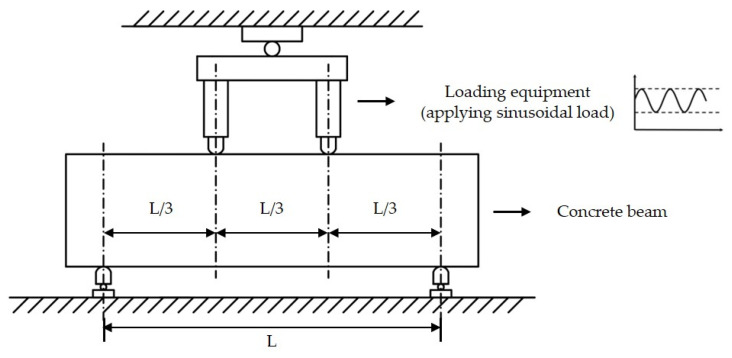
The schematic diagram of concrete beam fatigue test.

Different from the full-scale testing-based fatigue model, the concrete beam testing-based fatigue model was analyzed by the regression of the data from the laboratory concrete beam fatigue test. The test was performed by applying sinusoidal load at constant magnitude into the concrete beam directly, as shown in Figure 4. It is possible to clearly obtain the interior stresses’ change and structural performance deterioration of the concrete beam in a short time. The number of load repetitions to fatigue failure under different loading conditions can be obtained quickly, and it is easy to analyze the fatigue characteristic of cement concrete. It was shown that the relationship between the stress ratio (the ratio of stress to the modulus of rupture) and the number of load repetitions to failure is effective to describe the fatigue characteristics [1,34,35].

From 1922 to 1966, the Portland Cement Association (PCA) initially developed the fatigue model based on the regression analysis of the laboratory concrete beam fatigue test data. The fatigue model is shown in Equation (13) [36,37,38,39]. In Equation (13), PCA proposed the interior stress and the cumulative fatigue damage concepts. Furthermore, PCA assumed that a load with less than 50% of the flexural tensile stress had almost no effects on the fatigue deterioration of the concrete beam. This assumption was widely adopted by subsequent research.

(13) $$\log_{10}N=11.810-12.165\times \left(\frac{\sigma }{MR}\right)\;\;\;\;\;\;\;\mathrm{for}\;0.5<\frac{\sigma }{MR}<1.0$$

where:

N is the number of load repetitions to flexural failure of the concrete beam;
MR is the modulus of rupture;
σ is the stress at the bottom of the concrete beam.

Similarly, in the 1970s, the Federal Highway Administration (FHWA) proposed a zero-maintenance fatigue model, as shown in Equation (14) [40,41,42]. The data used in the fatigue model of FHWA were from the concrete beam tests conducted by Kelser, Ballinger, and Raithby from 1953 to 1974 [43,44,45]. For the fatigue testing of the concrete beam, the fatigue failure was defined as the fracture of the beam, and the stresses were calculated by the simple bending equation. The researchers recorded the data of the stress-to-strength ratio of the concrete beam under different loading conditions and the number of load repetitions for fatigue cracking. Based on the recorded data, the fatigue model was developed by the least square regression analysis. This fatigue model was employed in the pavement design procedure by the Illinois Department of Transportation in the 1990s.

(14) $$\log_{10}N=17.61-17.61\times \left(\frac{\sigma }{MR}\right)$$

where:

N is the number of load repetitions to flexural failure of the concrete beam;
MR is the modulus of rupture;
σ is the stress at the bottom of the concrete beam.

Based on the fatigue characteristics of concrete beams, researchers found that the range of stresses applied during the test also affected the fatigue strength of concrete [46,47,48]. It can easily be included that the R-value, which is the ratio of the minimum stress ($\sigma_{min}$) to the maximum stress ($\sigma_{max}$), should be involved in the fatigue model as well as the stress ratio. The fatigue model proposed by Aas-Jakobsen, which includes the R-value, is shown as follows [49]:

(15) $$\frac{\sigma_{max}}{f_{c}}=1-\beta \left(1-R\right)\log_{10}N_{f}$$

where:

$N_{f}$ is the number of load repetitions;
$\sigma_{max}$ is the maximum applied stress;
$f_{c}$ is the concrete strength;
β is an empirical coefficient with a value of 0.0640;
R is the ratio of the minimum stress to the maximum stress.

After that, Domenichini and Marchionna modified the empirical coefficient proposed by Tepfers to account for the differences between the laboratory test and real pavement structural conditions, including the influence of environment and the concrete slab properties [46,50]. However, it is challenging to introduce the R-value to the pavement design process due to the complexity of the stress fluctuations in the real pavement structure. The conclusions of the effects of stress fluctuations on the fatigue characteristics have not been widely used in airfield pavement design.

In China, the early research on fatigue models of cement concrete were mainly focused on highway pavements. The fatigue model was developed based on the relationship between the stress-to-strength ratio and the number of load repetitions obtained by laboratory concrete beam fatigue tests. The initial fatigue model in 1984 is a semi-logarithmic equation, as shown in Equation (16), and the fatigue test used for regression analysis was performed at an R-value of 0.1. Later, a large number of laboratory concrete beam fatigue tests with different stress ratios and stress range were conducted at Tongji University. The semi-logarithmic and double-logarithmic fatigue equations including stress ranges and stress-to-strength ratio were developed, as shown in Equations (17) and (18).

(16) $$S=\frac{\sigma_{max}}{f_{c}}=0.961-0.0631\log_{10}N_{f}$$

where:

S is the ratio of the maximum stress to the flexural strength;
$N_{f}$ is the number of load repetitions to fatigue failure;

(17) $$\log_{10}S=\log_{10}A-0.0422\left(1-R\right)\times \log_{10}N$$

(18) $$S=\frac{\sigma_{max}}{f_{c}}=B-0.0724\left(1-R\right)\times \log_{10}N$$

where:

R is the ratio of the minimum stress to the maximum stress.
A and B are the regression coefficients, and A is 1.0380 and B is 0.9993 when the probability of failure is 50%.

Afterward, the Civil Aviation Administration of China (CAAC) investigated the discrepancy between the fatigue characteristics reflected in the laboratory concrete beam fatigue tests and the airfield pavement structure. The fatigue model for airfield concrete pavement was developed based on the research into fatigue characteristics of highway pavement. Whether the fatigue strength concept proposed in 1995 or the allowable number of aircraft loading proposed in 2010, the essence of the fatigue model represents the relationship between the stress-to-strength ratio of concrete beam and the number of load repetitions [51,52]. The fatigue models from 1995 and 2010 are as follows:

(19) $$\frac{\sigma }{f_{cm}}=0.885-0.0631\times \log_{10}N_{e}\;\;\;\;\;\;\;\;\;\;\;\;\mathrm{in}\;1995$$

(20) $$\frac{\sigma }{f_{cm}}=0.9293-0.06615\times \log_{10}N_{e}\;\;\;\;\;\;\;\mathrm{in}\;2010$$

where:

$N_{e}$ is the number of load repetitions;
$f_{cm}$ is the flexural strength of concrete;
σ is the slab stress.

### 2.3. A Brief Summary

Based on the research above, it can be clearly found that the full-scale testing-based fatigue model is different from the concrete beam testing-based fatigue model in the process of regression analysis, the loading method, the definition of fatigue failure, and the stress calculation method. Table 2 shows the key factors that play an important role in the development of the two types of fatigue models. 

Summarily, the full-scale testing-based fatigue models were mainly based on the regression analysis of the full-scale test data. The loading was directly performed by applying the load of landing gear to the concrete slab. The fatigue failure was defined as 50% of the concrete slabs cracking, and the stress calculation methods vary from the Westergaard edge stress theory to the layered elastic analysis approach, and then to the finite element software. Comparably, the concrete beam testing-based fatigue models were mainly based on the laboratory concrete beam fatigue test. The concrete beams were directly applied the sinusoidal load at constant magnitude. The fatigue failure was defined as the bottom cracking of the concrete beams. The stresses were obtained with the bending equation of the simply supported beam.

**Figure 5:**
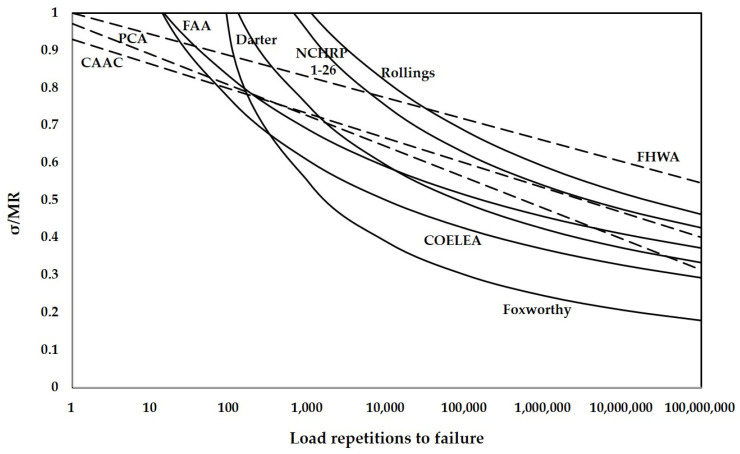
Fatigue curves of the fatigue models involved in this paper.

Different loading methods and definitions of fatigue failure contributed to the number of load repetitions to fatigue failure of concrete slabs or beams. Different stress calculation methods also had an impact on the determination of stresses in pavement structures. The distinctions of these factors led to a large difference in fatigue curve and design results, as shown in Figure 5. Due to the difference in fatigue curves, the designed pavement structures using different fatigue models under the same traffic loading would also have a large difference.

## 3. Comparison Analysis of Full-Scale Testing-Based and Concrete Beam Testing-Based Fatigue Models

### 3.1. Analysis Method

**Figure 6:**
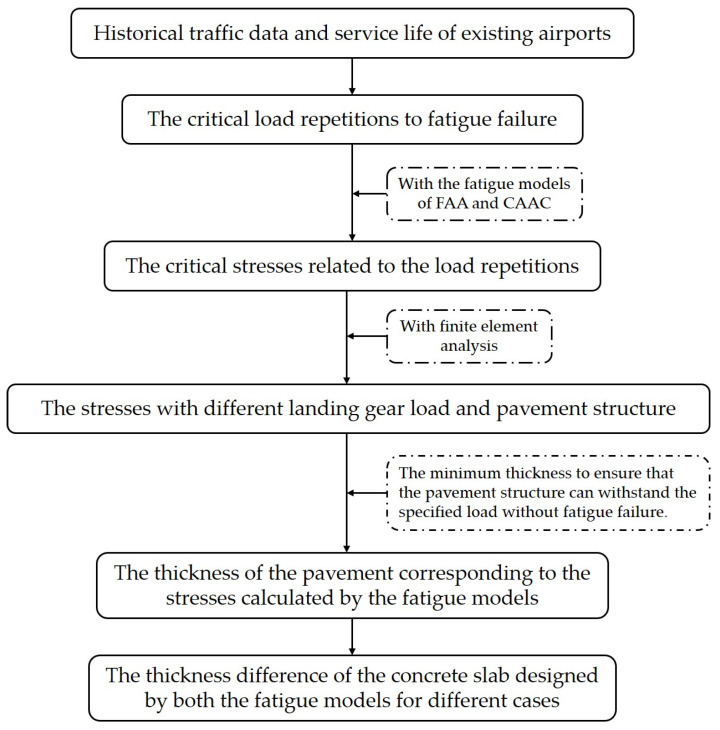
The flowchart of comparison analysis.

As mentioned above, it is meaningful to compare the existing fatigue models, especially for the two types of fatigue models: full-scale testing-based fatigue models and concrete beam testing-based fatigue models. In this paper, the full-scale testing-based model proposed by FAA Equation (11) was compared with the concrete beam testing-based fatigue model proposed by CAAC Equation (20). The thickness of the concrete slab was selected as the analysis parameter, due to its contribution to the fatigue resistance of the pavement structure. The procedure of the comparison analysis is shown in Figure 6.

**Figure 7:**
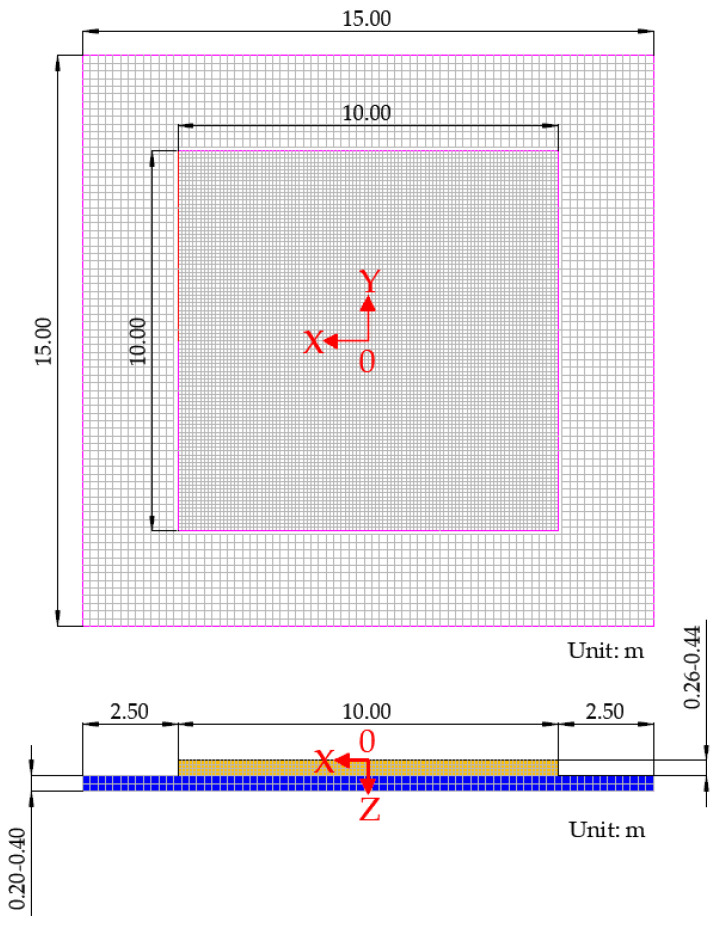
The three-dimensional finite element models developed in this paper.

In this paper, the three-dimensional (3D) finite element models (FEM) were established by the ABAQUS program shown in Figure 7. In the 3D FEM, the concrete slab and base layer were modeled as plates and the subgrade was modeled as Winkler foundation. The size of the concrete slab was set as 10 m by 10 m [53]. The width of the base extension was set to 2.5 m, meaning that the size of the base layer was 15 m by 15 m. The concrete slab and base layer materials were assumed to be isotropic and linearly elastic, whose properties were described by the modulus of elasticity and Poisson’s ratio. As for the contact interaction between the upper slab and base layer, the tangential behavior was assumed to be frictionless and the normal behavior was determined as hard contact [54]. For the boundary conditions, the normal displacements of the base layer on four sides were restrained. For the concrete slab, the displacements in both tangential directions on the opposite side of the load-acting side were restrained.

To balance the accuracy and speed of the calculation, the elements of both the upper slab and base layer were determined as eight-node linear brick elements with reduced integration (C3D8R) [55]. Besides, the sizes of the element for the concrete slab and base layer were set as 10 cm and 20 cm, respectively. The aircraft landing gear load, in the form of the static load, was applied at the critical load location of the concrete slab. The maximum principal stresses at the bottom of the slab were obtained for the comparison analysis. The parameters of structure and material properties used in the 3D FEM are presented in Table 3.

To investigate the effect of pavement structure and aircraft loading on the difference in fatigue models, different cases were analyzed. The parameters for different cases were assumed based on the traffic data of the existing airports and the actual pavement structure conditions. The specific values of the parameters are given in Table 4, and the loading parameters of the landing gears for selected typical aircraft are given in Table 5.

### 3.2. Results and Analysis

#### 3.2.1. The Influence of Load Repetitions

In this analysis, the thickness of the base layer is determined as 30 cm. The subgrade strength category is assumed as low strength (k = 40 MPa/m). The aircraft is chosen as B737-800. The number of load repetitions varies from 100,000 to 1,000,000. Accordingly, the calculated critical stress for fatigue models of CAAC and FAA are given in Table 6, respectively.

**Figure 8:**
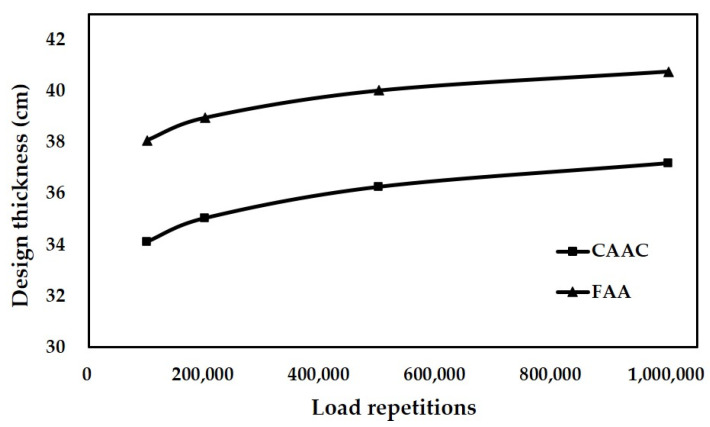
Designed thickness variation of concrete slabs with load repetitions.

According to the results of the critical stress, the thickness for different load repetitions can be determined, as shown in Figure 8. Figure 8 indicates that the design thickness increases with the number of load repetitions and the growth rate gradually slows down. The variation trend is similar to the fatigue curves shown in Figure 5, where the decrease in the stress-to-strength ratio is slowed down when the number of load repetitions keeps increasing. This is due to the design thickness of the slab being determined by the stresses calculated by the fatigue model. 

For the same number of load repetitions, the concrete slab designed with the fatigue model of FAA is thicker than that of CAAC. It can be clearly found that the fatigue model of FAA is more conservative than that of CAAC. In addition, the difference in thickness between the two models is always about 4 cm. With the increase in the number of load repetitions, the percentage value of the difference relative to the design thickness of CAAC continues to decrease due to the increase in slab thickness. Additionally, it can be seen that the absolute value of the difference between the design thickness of the two models is constant with the increase in load repetitions. This indicates that the two fatigue models are similar in their perception of the relationship between the slab thickness and the number of load repetitions. 

#### 3.2.2. The Influence of Aircraft Loading

**Figure 9:**
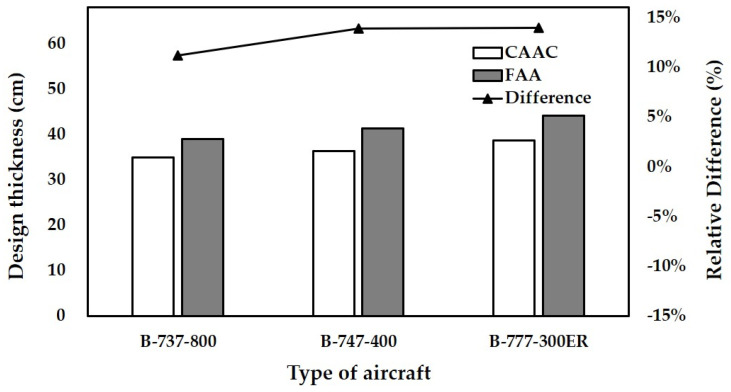
Designed thickness of concrete slabs for different aircraft.

In this analysis, the thickness of the base layer is determined as 30 cm. The subgrade strength category is assumed as low strength (k = 40 MPa/m). The number of load repetitions is assumed as 200,000. The aircraft varies from B737-800 to B777-300ER. The thicknesses of the concrete slab designed by the fatigue model of FAA and CAAC and the relative difference (the percentage value of the difference relative to the design thickness of CAAC) are shown in Figure 9, respectively.

From B737-800 to B777-300ER, both the thicknesses designed with the fatigue models of FAA and CAAC increase. The thickness differences also increase with the increase in aircraft loading. The influence of aircraft on the design thickness is mainly due to the different landing gear wheel loads. From B737-800 to B777-300ER, the landing gear wheel load is increasing, which leads to different damage caused by different aircraft in the pavement structure under the same load repetitions. Therefore, both the concrete slabs designed by the two fatigue models become thicker. Moreover, the thickness differences increase with the increase in wheel load. This indicates that the thickness of the concrete slab designed by the fatigue model of FAA is more conservative for larger aircraft.

#### 3.2.3. The Influence of the Thickness of Base Layer and Subgrade Strength

**Figure 10:**
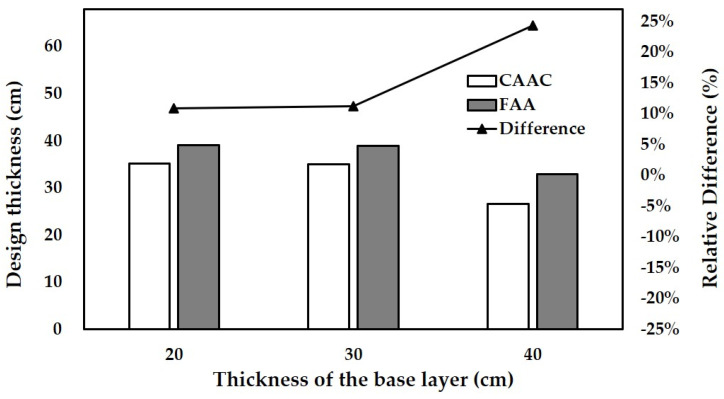
Designed thickness of concrete slabs for different thicknesses of the base layer.

**Figure 11:**
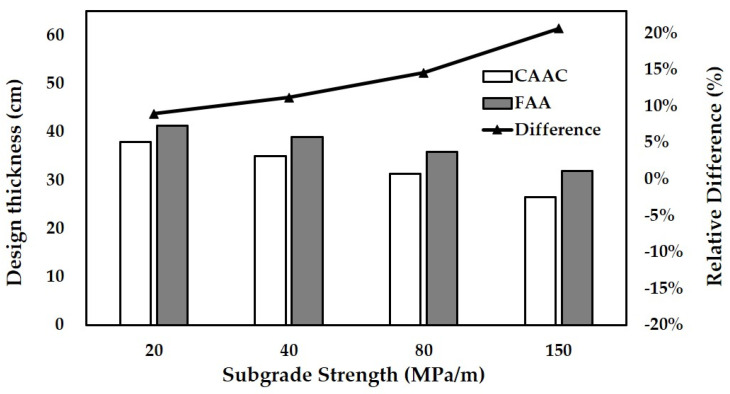
Designed thickness of concrete slabs for different subgrade strengths.

In this analysis, the number of load repetitions is assumed as 200,000. The design aircraft is assumed as B737-800. The thickness of the base layer varies from 20 cm to 40 cm. The subgrade strength varies from ultra-low strength to high strength. The thicknesses of the concrete slab designed with the fatigue model of FAA and CAAC for different thicknesses of the base layer are shown in Figure 10. The thicknesses for different subgrade strengths are shown in Figure 11.

With the increase in both the thickness of the base layer and the subgrade strength, the thicknesses of the concrete slab designed by the two fatigue models decrease, while the slab designed by FAA is always thicker than that designed by CAAC. It indicates that increasing the thickness of the base layer and the subgrade strength can be deemed as increasing the strength of the substructure beneath the concrete slab. The substructure can share a partial effect of the load. The increase in the strength of the substructure can improve the resistance to fatigue cracking of the overall pavement structure. Therefore, the thickness of the concrete slab designed by the two fatigue models presents a decreasing trend with the increase in base layer thickness and subgrade strength. Meanwhile, the difference in design thickness between the two fatigue models increases with the substructure strength increasing. This indicates that the fatigue model of FAA in pavement design would give a more conservative upper concrete slab when the strength of the substructure is high.

Moreover, the variation trend in Figure 11 can be mainly contributed to the different test processes of the full-scale testing-based fatigue model and the concrete beam testing-based fatigue model. A full-scale test is carried out by casting the concrete slab of the same size as the pavement structure on site. In the process of the full-scale test, the substructure beneath the concrete slab can share partial effects of the load, while in the concrete beam test, the concrete beam can resist all the fatigue cracking induced by the load repetitions. The difference in the test process results in the difference of the thickness of the concrete slab designed by the two fatigue models. With the increase in substructure strength, the substructure would have a larger effect to resist fatigue cracking in the fatigue model of CAAC model compared to that of FAA, which leads to a larger reduction in the thickness of the concrete slab. Thus, the design thickness of FAA is more conservative than that of CAAC as the strength of the substructure increases.

## 4. Recommendations for Fatigue Models of Airfield Concrete Pavements in the Future

For the fatigue model proposed by FAA, most of the data used in the regression analysis were conducted by COE in Lockbourne, Sharonville, and other sites from 1943 to 1973. Since the historical full-scale tests were conducted a long time ago, the test conditions and pavement structure were limited by the research level and the aircraft load, as well as the pavement structure at that time. Therefore, the fatigue characteristics of the concrete structure reflected by the historical full-scale data could not be well fitted to the current airfield concrete pavement. Further, the fatigue model proposed by CAAC was mainly obtained by the concrete beam fatigue test. Compared to the concrete beams, the stresses of real pavement structures are more complex. In the operation, the factors, such as temperature and humidity, would have a certain impact on the fatigue characteristics of the concrete slab.

Therefore, the current fatigue models cannot be widely and effectively used for the design of airfield concrete pavement structures due to the limitations of their historical test data. Based on the previous research of the development process of the fatigue model, the future fatigue model can be improved from the following three aspects:

- Data source.

The development of fatigue models in the future should be based on historical full-scale test databases supplemented with the local test data in different regions. However, it is costly in terms of manpower and investment to conduct full-scale tests in different regions, considering multiple influencing factors, such as loading method, pavement structure, and environmental conditions. Luckily, many field evaluations on airfield pavements have been performed and a great deal of evaluation data have been collected. Therefore, the Structural Condition Index (SCI) and the related air traffic data from the evaluated pavements can be added to the historical database. It can solve the problem that the fatigue characteristics reflected by the historical database do not match the local airport pavement structure during the development of fatigue models.

- Stress calculation method.

Historical stress calculation methods have developed from Westergaard edge stress theory to layered elastic analysis and then to finite element analysis. It can be seen that the development of stress calculation methods is a process of progressively more accurate calculation of stresses in pavement structures. The process of developing fatigue equations in the future is suggested to be based on numerical simulations by finite element analysis. The accuracy and efficiency of stress calculation can be improved by adjusting the conditions and parameters of the finite element model. In the future, artificial intelligence algorithms, such as artificial neural networks (ANNs), can be introduced in the structural stress calculation of cement concrete [56,57,58]. A database of stress analysis can be developed through field tests and numerical simulations, after which the artificial intelligence algorithm model can be trained based on the database to achieve stress prediction.

- Regression analysis process

After obtaining data that are sufficient to reflect the fatigue characteristics of the local pavement structure, the SCI data from different periods can be linearly fitted to the logarithm of coverages, for which has been observed by Rollings that SCI deteriorates as a linear function of the logarithm of coverages [29]. After linear regression analysis, the coverages when the pavement structure starts to crack (when SCI deteriorates from 100) and the coverages to fatigue failure (when SCI equals 0) can be obtained. These data and stress-to-strength ratios will be supplemented to the historical database for re-regression analysis in order to obtain the modified coefficients for the improved fatigue model [28,59]. During the process, if the structural conditions of the pavement structures are similar in different testing periods, resulting in obtained data that cannot be regarded as valid being added to the historical database, it is suggested that the fatigue model be developed by conducting full-scale tests and regression analysis locally in different regions.

The improved method of the fatigue model based on the three aspects mentioned above is proposed in this paper. It can help develop fatigue models to be more suitable for the local pavement structures, based on the previous studies, in the future. By applying the improved fatigue model to the design of pavement structure, the fatigue resistance of the pavement structure will be enhanced and the risk of fatigue cracking could be reduced. Meanwhile, it is also useful to estimate more accurately the service situation and remaining life of the concrete structure in the evaluation of the pavement.

## 5. Conclusions

1. According to the regression analysis process, the fatigue models are divided into two types: the full-scale testing-based fatigue model and the concrete beam testing-based fatigue model. This paper reviews the development process of two types of fatigue models for airfield concrete pavement. It can be clearly found that the full-scale testing-based fatigue model is different from the concrete beam testing-based fatigue model in the process of regression analysis, the loading method, the definition of fatigue failure, and the stress calculation method.
2. Compared with the fatigue model of CAAC, the fatigue model of FAA always tends to design a more conservative and safer thickness of concrete slab with the same load repetitions in different cases, thus making the pavement structure have sufficient resistance to fatigue cracking. The difference between the thickness of the two models is less influenced by the number of load repetitions but largely affected by factors such as aircraft type, the thickness of the base layer, and subgrade strength. This is caused by the different test methods and test conditions between the two fatigue models.
3. Moreover, due to the limitations of the historical test data, the current fatigue models cannot be applied to various pavement structures in different regions. Therefore, this paper proposes an improved method to refine the future fatigue model from three aspects: data source, stress calculation method, and regression analysis process.

## Figures and Tables

**Table 1:** The full-scale test data used for the regression analysis in the fatigue model of FAA.

Test Sites	Number of Data Points
Lockbourne No. 1	15
Lockbourne No. 2	3
Sharonville Heavy Load Tests	1
Multiple Wheel Heavy Gear Load (MWHGL) Tests	4
Keyed Longitudinal Joint Study (KLJS)	4
Soil Stabilization Pavement Study (SSPS)	3
National Airport Pavement Test Facility (NAPTF)	7
Total	37

**Table 2:** Summary of fatigue models for airfield concrete pavements [4].

Types	Fatigue Model	Regression Data	Stress Type	Failure Definition	Stress Calculation Method
Full-scale testing-based fatigue models	COE (1946)	COE field data	Load only	50% of slabs cracking	Westergaard edge stress theory
	Improved COE (1957)	COE field data	Load only	50% of slabs cracking	Westergaard edge stress theory
	COE-LEA (1979)	COE field data	Load only	50% of slabs cracking	Layered elastic analysis
	Rollings (1990)	COE field data	Load only	50% of slabs cracking	Layered elastic analysis
	Foxworthy (1985)	COE field data	Load only	50% of slabs cracking	Finite element (ILLI-SLAB)
	Darter (1990)	COE field data	Load only	50% of slabs cracking	H-51
	NCHRP 1-26 (1992)	COE field data & AASHO road test data	Load and temperature curling	50% of slabs cracking	Finite element (ILLI-SLAB)
	FAA (2010)	COE field data & NAPTF data	Load only	50% of slabs cracking	Finite element (3D-FE)
Concrete beam testing-based fatigue models	PCA (1963)	Concrete beams	Load only	Beam fracture	Beam bending equation
	Aas-Jakobsen (1970)	Concrete beams	Load only	Beam fracture	Beam bending equation
	FHWA (1977)	Concrete beams	Load only	Beam fracture	Beam bending equation
	CAAC (1995&2010)	Concrete beams	Load only	Beam fracture	Beam bending equation

**Table 3:** The parameters of pavement structure and material properties used in the FEM.

Pavement Structure	Parameters	Values
Concrete slab	Size of plate	10 m × 10 m
	Elasticity modulus	29 GPa
	Poisson’s ratio	0.15
	Elements	C3D8R
	Element size	10 cm
Base layer	Size of plate	15 m × 15 m
	Elasticity modulus	2000 MPa
	Poisson’s ratio	0.20
	Elements	C3D8R
	Element size	20 cm

**Table 4:** Variation parameters of analyzed cases in this paper.

Parameters	Values
Load repetitions	100,000, 200,000, 500,000, 1,000,000
Aircraft types	B737-800, B747-400, B777-300ER
Thickness of base layer	20 cm, 30 cm, 40 cm
Subgrade strength	High strength subgrade (k = 150 MPa/m) ^1^, Medium strength (k = 80 MPa/m), Low strength (k = 40 MPa/m), Ultra-low strength (k = 20 MPa/m)

^1^ The k-value represents the modulus of subgrade reaction.

**Table 5:** Loading parameters of the landing gears for selected typical aircraft.

Aircraft	Single Wheel Load (kN)	Tire Pressure (MPa)	Number of Landing Gears	Number of Wheels	Axle Spacing (m)	Wheel Spacing (m)
B737-800	187.6	1.40	2	2	-	0.86
B747-400	236.2	1.38	4	4	1.12	1.47
B777-300ER	265.3	1.50	2	6	1.40	1.45/1.48

**Table 6:** The critical stresses for different load repetitions.

Load Repetitions	Critical Stress, MPa	
	CAAC	FAA
100,000	3.990	3.420
200,000	3.857	3.294
500,000	3.682	3.141
1,000,000	3.549	3.035

## Data Availability

Not applicable.
